# Transcription and Maturation of mRNA in Dinoflagellates

**DOI:** 10.3390/microorganisms1010071

**Published:** 2013-11-01

**Authors:** Sougata Roy, David Morse

**Affiliations:** Institut de Recherche en BiologieVégétale, Département de Sciences Biologiques, Université de Montréal, 4101 Sherbrooke east, Montréal, QC H1X 2B2, Canada; E-Mail: sougatairbv20@gmail.com

**Keywords:** transcription factor, gene expression, regulation

## Abstract

Dinoflagellates are of great importance to the marine ecosystem, yet scant details of how gene expression is regulated at the transcriptional level are available. Transcription is of interest in the context of the chromatin structure in the dinoflagellates as it shows many differences from more typical eukaryotic cells. Here we canvas recent transcriptome profiles to identify the molecular building blocks available for the construction of the transcriptional machinery and contrast these with those used by other systems. Dinoflagellates display a clear paucity of specific transcription factors, although surprisingly, the rest of the basic transcriptional machinery is not markedly different from what is found in the close relatives to the dinoflagellates.

## 1. Introduction

Dinoflagellates are an important group of unicellular eukaryotes found in both marine and fresh water environments. These marine species are of particular importance on a global scale, as along with the diatoms, they contribute roughly half of the carbon fixed in the oceans, and thus roughly a quarter of the global totals [1]. They also play a role in maintaining the biodiversity surrounding coral reefs, since the coral polyps themselves rely on photosynthetic products supplied by the symbiotic dinoflagellates they harbor for growth in nutrient poor waters [2]. Furthermore, many marine dinoflagellates synthesize potent toxins that accumulate to high concentrations in the algal blooms commonly called “red tides” [3]. Lastly, the nightly bioluminescence of many dinoflagellates, popularly known as the “phosphorescence of the sea”, has inspired not only art and literature but also intensive scientific dissection of the bioluminescence phenomenon [4]. Interestingly, in *Lingulodinium polyedrum* this nightly bioluminescence [5], as well as photosynthesis [6], cell division [7], and diurnal vertical migration [8], are all regulated by an endogenous circadian (daily) clock. *L. polyedrum* has been studied for over 60 years as a model system for addressing the biochemical links between the internal clock and the observed rhythms [9].

**Figure 1:**
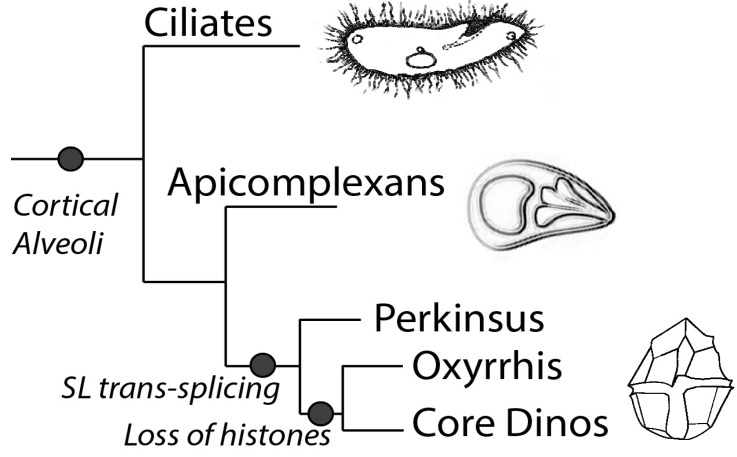
The diagram shows the schematic representation of the phylogeny of the superphylum Alveolata, which is marked by the presence of the cortical alveoli. Splice leader *trans*-splicing is a common feature in all the members of the dinoflagellate clade, while *Oxyrrhis* and the core dinoflagellates lack histones and have a dinokaryotic nucleus.

Phylogenetically, dinoflagellates are grouped in the superphylum Alveolata, which contains apicomplexans as their closest relatives as well as ciliates [10]. Members of the Alveolata share a number of features, in particular the presence of flattened vesicles termed cortical alveoli lying just beneath the plasma membrane (Figure 1). However, dinoflagellates also have many unique characteristics compared to their relatives. For example, dinoflagellates typically possess a large quantity of nuclear DNA containing many genes organized in tandem gene arrays, with DNA found in a liquid crystal structure lacking observable nucleosomes [11]. It is unfortunate that dinoflagellates have so far proven refractory to mutational or gene transformational studies, thus hindering the extensive molecular studies needed to understand the mechanisms for regulating gene expression.

The mechanisms used to control the expression of different genes have been extensively researched in both prokaryotes and eukaryotes. Critical events in eukaryotes include changes in chromatin organization, transcription of DNA into pre-mRNA, splicing of pre-RNA into mature mRNA, mRNA transport, mRNA degradation, mRNA editing and covalent modifications of the mRNA, translation of mRNA into protein, and, lastly, post-translational modification of the protein. All these, either individually or collectively, are responsible for regulating gene expression within a cell. In this review, we will focus primarily on transcription and its regulation as they relate to the control of gene expression in the dinoflagellates, as more comprehensive studies on dinoflagellates have been published elsewhere [12,13,14].

## 2. Transcription and Its Regulation

### 2.1. *cis*-Acting Sequences and RNA Polymerase Components

**Figure 2:**
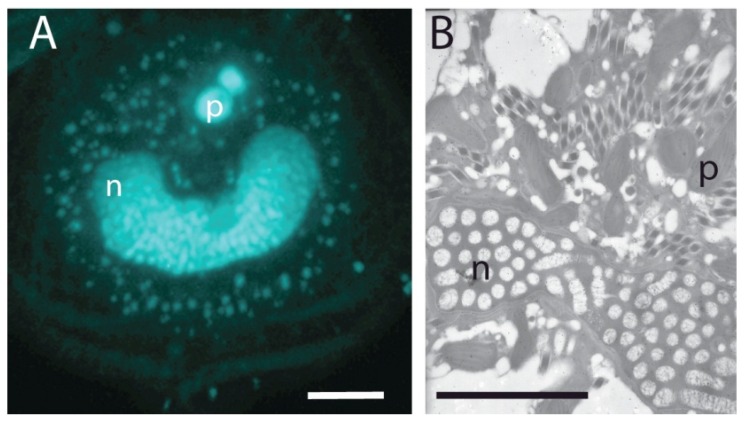
(**A**) Permanently condensed chromosomes of the dinoflagellate *Lingulodinium polyedrum* (the cultures were obtained from the National Center for Marine Algae, Maine) as visualized by fluorescence microscopy after DAPI. The C-shaped nucleus (n) is surrounded by the small punctate DNA staining of the multiple plastid genomes and lies under two larger spherical PAS bodies (p) at the apical end of the cell. (**B**) The nucleus viewed by transmission electron microscopy. The cross section shown lies near the back of the C-shaped nucleus (n) and shows chromosomes cut both in cross section (ovals) and longitudinally (cylinders), as well as plastids (p) and numerous diamond-shaped trichocysts. All scale bars are 10 µm.

Dinoflagellate chromosomes are permanently condensed at all stages of the cell cycle (Figure 2) and assume a liquid crystalline structure [15,16] with bivalent cations acting as the stabilization matrix [17]. This unusual chromatin structure thus raises the important questions about the accessibility of genes within the structure to the transcriptional machinery. The dinoflagellate *Prorocentrum micans* was inspected using high resolution electron microscope autoradiography for ^3^H-adenine incorporation, and this revealed that RNA transcription was prevalent only on extrachromosomal DNA filaments and not on DNA within the main body of the chromosome [18]. It was proposed that this transcriptionally inactive DNA might instead play a role in stabilizing chromosome organization, perhaps by an association with a protein matrix [18].

Given access to the genetic material, transcription initiation in dinoflagellates is likely to require an elaborate set of *trans*-acting factors and a series of conserved *cis*-acting sequences, as is the case in other eukaryotes. The complex of *trans*-acting factors binding the regulatory sequences in the DNA includes, in addition to the RNA polymerases, both general and gene-specific transcription factors, activators and mediators [19]. The *cis*-acting sequences in eukaryotes can include regulatory elements far from the transcription start site, termed enhancers, although the region just upstream of the start site, termed a promoter, consisting of a core region and other regulatory domains [20,21] is considered as the primary site of initiation. There are two major classes of promoters that regulate the expression of protein coding genes, and these contain either a TATA-box (consensus sequence <TATAAA>) or CpG islands, a region rich in CG dinucleotides [22] as their core domains. In *Pyrocystis lunula* luciferase (*lcf*) genes, a GC box consensus sequence <GGGCGG> is present, but its location is further upstream than the usual position of −110 (numbered relative to the transcriptional start site at +1) found in many eukaryotes [23]. Furthermore, a GC-rich motif <C(G/C)GCCC> was also found within the upstream region of *P. lunula lcf A* and *L. polyedrum lcf* and *lbp* genes, but its position was not fixed. This GC-rich motif was first reported in the upstream region of the *Peridinium bipes* ferredoxin gene [24]. However, the role of this motif in gene expression has still not been established. Both TATA-box or CpG island type promoters may include additional sequence elements such as the GC-box <GGGCGG>, the CAAT-box <CCAAT>, and the INR box <(C/T)(C/T)AN(T/A)(C/T)(C/T)> at which transcription is initiated. Interestingly, the TATA box is quite conserved in eukaryotes and is also found in protists as diverse as amoebas (*Acanthamoeba*), slime molds (*Dictyostelium*), ciliates (*Histriculus cavicola*), and apicomplexans (*Plasmodium*) [25,26,27,28,29,30]. On the other hand, members of the phylum Parabasalia use their own specific promoter element instead of the canonical TATA box [31,32,33].

Proper understanding of gene organization and structure is required to describe transcription in dinoflagellates. For example, *L. polyedrum* has multiple copies of peridinin-chlorophyll *a*-binding protein (*pcp*), luciferin binding protein (*lbp*) and luciferase (*lcf*) genes arranged in long tandem repeats [34,35,36,37]. PCR with *Pyrocystis lunula* genomic DNA revealed that, among *lcf* *A*, *lcf* *B* and *lcf* *C* isoforms, two (*lcf* *A* and *B*) are in tandem repeat. However, the sequence of the intergenic region between *lcf* and *pcp* coding sequences of the *L. polyedrum* lacks any known promoter elements. The only common feature between the two was a conserved 13 nucleotide sequence, CGTGAACGCAGTG, proposed as a dinoflagellate specific promoter sequence [35] but no further work has been published to firmly establish this result. Moreover, this sequence is not conserved among different dinoflagellate species as it is absent in the intergenic region between *P. lunula lcf* *A* and *lcf* *B* genes [38]. To test if the tandem repeat structure is a general character of dinoflagellates, PCR was used with primers directed away from one another in *Amphidinium carterae* [39]. PCR using genomic DNA as a template was expected to produce a band if the genes were found as a tandem repeat, and this strategy revealed that 17 out of the 47 genes tested did indeed have a tandem repeat structure.

The lack of identifiable sequence elements in the intergenic spacers has lead to the suggestion that tandem gene repeats may form a polycistronic transcript, in a manner similar to the *Trypanosoma* gene structure [40]. The trypanosomes transcribe from a single promoter long polycistronic transcripts containing genes coding for different gene products, and the primary transcript is then processed into mature mRNAs by *trans* splicing of the SL leader at the 5′ end and by polyadenylation at the 3′ end. If true for dinoflagellates, one possibility would place a promoter upstream of each tandem array, thus explaining the lack of recognizable promoter sequences in the intergenic regions. However, the consequences of this hypothesis include the predictions that the intergenic spacer region should be abundant in the transcribed RNAs, and that sequence differences between copies in low copy number arrays should be detected in the mature transcripts at a frequency inversely proportional to the copy number. In a recent transcriptomic study that addressed this issue, none of these predictions were validated experimentally [41].

**Figure 3:**
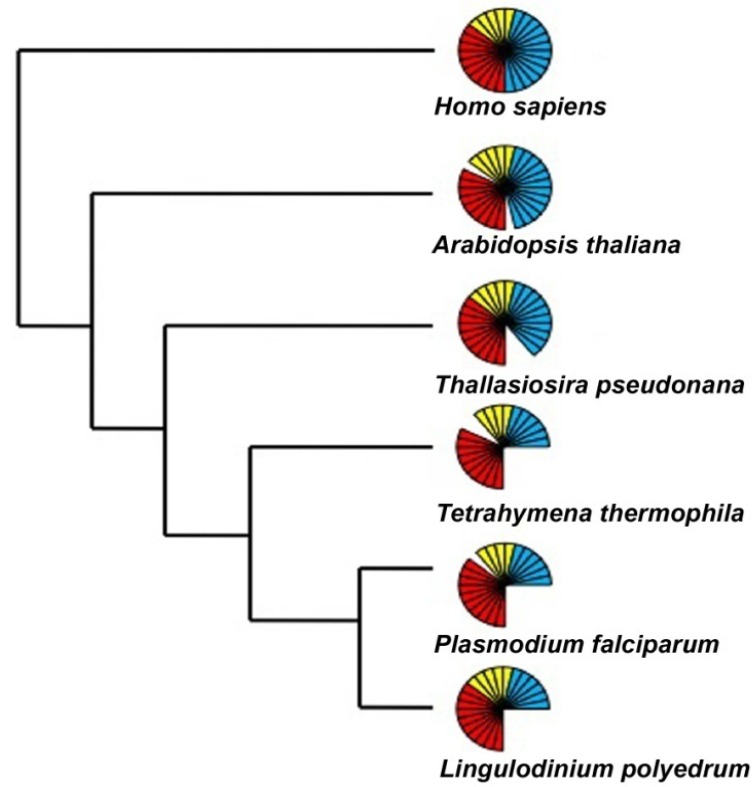
The number of RNA polymerase components present over a wide phylogenetic range of organisms includes those considered to be core components (red), common components (yellow) and specific components (blue) of the RNAP I, II and III. Each bar represents an individual component. The representative sequences for the RNA polymerase I, II and III subunits were selected from an animal (*H. sapiens*), a plant (*A. thaliana*), a diatom (*T. pseudonana*), and two other alveolates (*T. thermophila* and *P. falciparum*) and uploaded and maintained as a local database in the Geneious software. Using tBLASTn and an expect E-value cutoff of e^−25^, the *Lingulodinium* transcriptome was scanned to obtain the homologues for the RNA polymerase subunits [41]. For all other species the sequences were directly obtained from the KEGG specific pathway database by selecting the specific organism.

Eukaryotic and prokaryotic transcription also differs in that three different RNA polymerases (RNAP) are used for the former while only one is used for the latter. The three eukaryotic enzymes have specialized functions, with RNAP I transcribing most ribosomal RNA (rRNA), RNAP II transcribing protein-coding messengers (mRNA), small nuclear RNAs (snRNA) and micro RNA (miRNA), and RNAP III synthesizing transfer RNAs (tRNA) and the 5S rRNA. An assessment of the activity of RNA polymerase in the dinoflagellate *Crypthecodinium cohnii*, carried out with radiolabeled UTP, revealed that considerable amounts of RNA polymerase activity remained even after inhibition by α-amanitin, a potent inhibitor of RNAP II. This thus confirmed the presence of multiple forms of DNA dependent RNA polymerase as in other eukaryotes [42]. Curiously, this research also noted a peculiar inhibition of polymerase activity by Mn^+2^, instead of the activation of these enzymes seen in other eukaryotes. It was suggested that dinoflagellate RNAP II activity might differ slightly from the other eukaryotic RNAP II enzymes [42], perhaps analogous to the unusual form of RNAP II found in some trypanosomes [43]. However, the transcriptome of *L. polyedrum* contains a complete set of core and common elements for all the three eukaryotic RNAPs. Furthermore, the specific elements absent from the transcriptome were also missing in other members of the Alveolata (Figure 3). It seems that the alveolates in general can assemble functional RNAPs with a reduced number of components as compared to higher eukaryotes, and there is nothing unique to the dinoflagellates in this part of the transcriptional machinery.

### 2.2. Basal/General Transcription Factors

**Figure 4:**
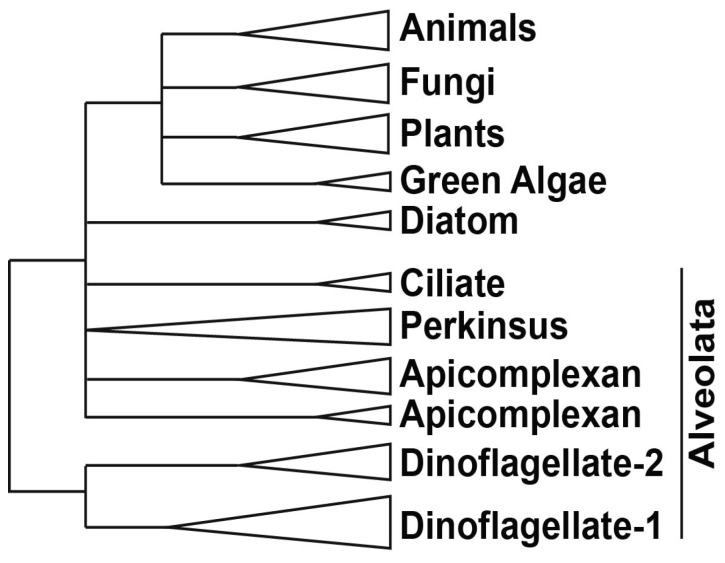
A simplified phylogeny of TBP and TBP-like proteins shows that the two TBP-like clades unique to dinoflagellates are distinct from all other TBP clades. The apicomplexan TBPs also form two clades, one from proteins in *Babesia* and *Toxoplasma* and the other from proteins in *Cryptosporidium* and *Plasmodium*. The protein sequences used include: animals—*Homo sapiens* (CAG33057.1), *Mus musculus* (AAH50136.1), *Gallus gallus* (BAA20298.1), *Xenopus laevis* (NP 001084369.1), *Danio rerio* (AAQ07596.1), *Drosophila melanogaster* (AAA79092.1), *Strongylocentrotus purpuratus* (NP_999786.1); plants—*Arabidopsis thaliana* (AEE75356.1), *Oryza sativa* (ABA99084.1), *Glycine max* (NP 001238202.1), *Zea mays* (NP 001105318.1); green algae—*Volvox carteri* (XP_002948268.1), *Chlamydomonas reinhardtii* (XP_001691004.1); diatoms—*Phaeodactylum tricornutum* (XP_002186321.1), *Thalassiosira pseudonana* (XP 002293666.1); fungi—*Neurospora crassa* (XP_960219.1), Candida tropicalis (XP 002548983.1), *Aspergillus nidulans* (XP_662580.1); alveolata—*Cryptosporidium muris* (XP 002139943.1), *Cryptosporidium parvum* (AAR21861.1), *Tetrahymena thermophila* (EAR92317.1), *Toxoplasma gondii* (XP_002368492.1), *Ichthyophthirius multifiliis* (XP_004031283.1), *Babesia bovis* (XP_001610545.1), *Plasmodium vivax* (EDL43506.1), *Plasmodium falciparum* (XP_001351620.1), *Perkinsus marinus* (XP 002782410.1), (XP 002782409.1) and (XP 002782411.1), *Crypthecodinium cohnii* (AAL24503.1), *Lingulodinium polyedrum* (JO752877.1) and (JO755256.1), *Symbiodinium* (kb8 c12831), (kb8 c27940), (mf105 rep c7144), (mf105 rep c14572) and (mf105 rep c49191) [56]. For *L. polyedrum* and *Symbiodinium*, the translated sequences were aligned using MUSCLE, an alignment program built in the tree construction software MEGA5 [57] that was used for this phylogenetic analysis.

In addition to RNAP II, an *in vitro* reconstitution of a functional eukaryotic transcriptional apparatus requires a suite of other basal/general transcriptional factors (TF) [44]. Six multi-subunit complexes, termed TFIIA, TFIIB, TFIID, TFIIE, TFIIF and TFIIH, appear to be among the most important [45,46,47,48,49]. The first step of promoter recognition is performed by TFIID, constituted from the TATA binding protein (TBP) and at least 14 TBP-associated factors (TAFs) [50,51]. TBP binding is considered to be the rate-limiting step in the transcription process [52], although TBP can have relatives, such as TBP-related factors (TRF), which also activate transcription from the same RNAP II promoters that are activated by TBP [53,54]. These TRFs have been found in diverse animals, including fruit fly, nemotode, frog, zebrafish, chick, mouse, and human [53]. Interestingly, *C. cohnii* has been shown to contain a TBP-like factor (TLF), clearly homologous to TBP yet lacking the four phenylalanine residues known to interact with the TATA box. This TLF is unique to dinoflagellates (Figure 4, Supplementary Figure S1) and has a strong affinity for a <TTTT> sequence instead of the consensus TATA-box sequence [55]. Unfortunately, the upstream regions from 6 different genes of two different dinoflagellates did not contain a TTTT element [55]. This suggests a unique promoter recognition mechanism for at least these genes, in keeping with the unusual structure of the chromatin of these organisms.

**Figure 5:**
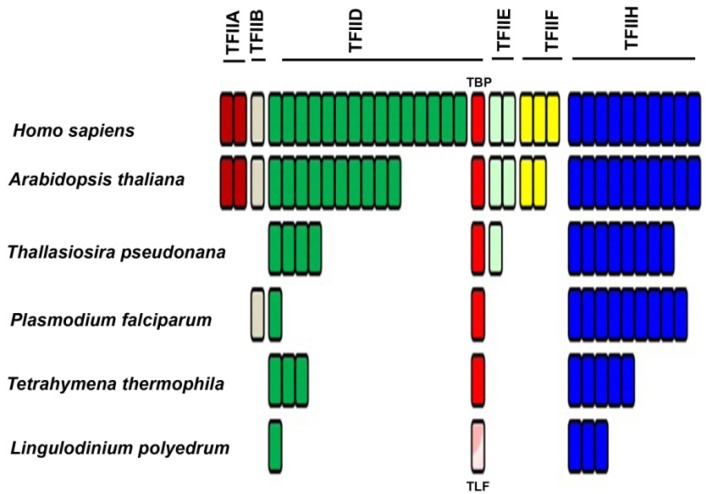
Phylogenetic distribution of transcription factors associated with RNA-polymerase II shows a marked decrease in the number of TFII members among the apicomplexans. The dinoflagellates do not contain the putative TBP (red) but do express a TBP-like factor (TLF; pink). Each bar represents a different component. A pool of basal transcription factor (BTF) protein sequences were selected from the five species then stored as a local database in Geneious. The *Lingulodinium* transcriptome was scanned using tBLASTn at an expect E-value of <e^−25^, to obtain the homologues of the BTFs [41].

The *L. polyedrum* transcriptome contains two TLF isoforms similar to the TLF found in *C. cohnii* and, somewhat surprisingly, no TBP at all [41]. The phylogenetic relationship between the consensus TBP and the TLF, found uniquely in the dinoflagellates, clearly indicates the early divergence of TLF from TBP as well as the presence of two distinct TLF clades within the dinoflagellates (Figure 4). In agreement with this lack of TBP, it is perhaps not surprising that *L. polyedrum* also lacks most other TAFs, although the closely related *Alexandrium* expresses two proteins with DNA helicase activity, RuvB-like1 and RuvB-like2 [58]. RuvB-like proteins have been shown to co-purify with the human RNA polymerase holoenzyme complex and found to be an extremely important element required for growth [59], suggesting they may also play a role in the dinoflagellates. In particular, *L. polyedrum* lacks any TFIIA, TFIIB, TFIIE or TFIIF components, and only 3 out of the ten expected TFIIH components are found (the E-value cut-off for the tBLASTn is e^−25^). It must be noted, however, that ciliate, apicomplexan and diatom genomes contain a single TBP and also lack the TAFs and TFs missing in *L. polyedrum* [41]. A figurative representation of basal TF status in different eukaryotes (Figure 5) indicates that the poor conservation of TAFs and other basal TFs in *L. polyedrum* is commensurate with the other related eukaryotes. These properties thus seem more likely to be due to a reduced dependence on these TFs throughout the Alveolata than to the unusual nature of the dinoflagellate chromatin.

### 2.3. DNA Binding Proteins

Histones are the most abundant and conserved class of basic proteins in the DNA-binding protein class of eukaryotes, and can profoundly affect transcription rates by their ability to alter the degree of chromatin condensation. The classic nucleosome structure, observed microscopically as “beads on a string”, forms when 146 bp of DNA wraps 1.65 times around the histone octamer (dimers of each of the four core histone proteins H2A, H2B, H3, and H4) [60,61]. A fifth protein, histone H1, binds to the linker DNA between nucleosomes to induce an even higher structural order to the chromatin [62]. Dinoflagellates have long been thought to lack histone proteins, and there is considerable biochemical evidence to support this view [63]. Dinoflagellate protein extracts do not show the typical pattern of histones after polyacrylamide gel electrophoresis [64,65] and no nucleosomes are visible in dinoflagellate DNA spreads observed under a microscope [66,67]. The only other eukaryotic cells lacking histones are sperm nuclei, which instead employ arginine-rich proteins called protamines to stabilize their DNA structure [15,68,69]. No protamines are found in the *L. polyedrum* transcriptome.

Although metatranscriptomic analysis with the DinoSL found core histone sequences that were scattered in different dinoflagellate species [70], the presence of the full complement of all core histones in a single dinoflagellate species were first confirmed in *L. polyedrum* [71]*.* However, the presence of all the core histone sequences in the transcriptome of two dinoflagellate species, the high sequence conservation of these sequences compared to other eukaryotic histones, and the presence of a wide range of histone modifying enzymes in the *L. polyedrum* transcriptome all suggest that histone proteins are indeed expressed [56,71], albeit at levels still undetectable by antibody or MS analysis [71].

The total amount of basic proteins in dinoflagellate nuclei (basic protein to DNA ratio of 1:10 [72]) is much lower than generally found in eukaryotes (1:1 ratio [73]) and prokaryotes (1:1.75 ratio [74]) and appears to date to include two different basic protein types. One, a group of histone-like proteins (HLPs) [65], were first found by electrophoretic analysis of acid soluble nuclear proteins in the dinoflagellate *C. cohnii* and later renamed HCc 1–4 [75,76]. Blast homology searches with *C. cohnii* HLP revealed that *L. polyedrum* also has an HLP, which was named HLp [77], and this protein was shown to have sequence specific DNA binding activity and be subject to post-translational modifications suggesting that its activity might be regulated *in vivo* [77]. The presence of HLPs has been confirmed in many other dinoflagellates [63]. A second basic protein called DVNP (dinoflagellate/viral nucleoprotein), recently found in studies of the basal dinoflagellate, *Hematodinium*, can bind DNA as efficiently as histones and can also be post-translationally modified [78]. DVNP is found only in dinoflagellates, including the early diverging lineage *Hematodinium*, as well as in a family of large algal virus, the Phycodnaviridae. However, DVNP is not found in *Perkinsus*, the common ancestor of dinoflagellates and apicomplexans, which has instead the typical eukaryotic chromatin with all core histone proteins and DNA arranged into nucleosomes [79]. The acquisition of DVNP thus occurred at some time following divergence of *Hematodinium* and the main dinoflagellate lineages from *Perkinsus*, and thus appears to coincide with the appearance of the unusual core dinoflagellate nuclear morphology. In addition, a substantial proportion of the DNA appears to consist of repeated sequences, and it is possible that this may contribute to genome organization [80].

The nuclear matrix is a network of fibers in the nucleus that also plays a key role in the functional and structural organization of the chromatin. Electron microscopy studies of nuclear matrices in the dinoflagellate *Amphidinium carterae*, produced *in situ* by microencapsulation in agarose and sequential extraction coupled with immunoblotting, revealed the presence of two matrix proteins (lamins and topoisomerase II) similar to what is found in higher eukaryotes [81]. The lamins are architectural proteins, a class of intermediate filaments that line the inside of the metazoan nuclear envelope and act as a scaffold to which proteins and chromatin bind [82]. They have a wide range of nuclear functions such as higher-order genome organization, chromatin regulation, transcription, DNA replication and repair [83,84]. Thus, although the dinoflagellate chromatin is arranged differently from other eukaryotes, its nuclear matrix is conserved, perhaps indicative of an ancient evolutionary trait required for nuclear structure.

In pursuit of sequence-specific DNA binding proteins (as opposed to basal or general TFs), a dinoflagellate nuclear associated protein (Dinap1) was found in *C. cohnii*. Dinap1 does not have any known homologues but does contain two zinc finger domains (known to be present in many transcriptional factors) and two WW domains (known to interact with proline-rich domains) [85]. An interaction study using the Dinap1 WW domains identified five proline-rich Dinap1-interacting proteins (Dip) [86], and screening of a *C. cohnii* cDNA library with a tagged Dip1 retrieved not only the expected Dinap1 but also other interactants, named DAP (Dip1-associated proteins) [86]. Dinap1, Dip1 and DAP were all found in the nucleus and all have the same pattern of protein expression. Unfortunately, none of the above-described proteins interacted with DNA directly [86], although some as yet unidentified intermediate partners may be involved in DNA recognition. In addition to Dinap1, a homologue of the Tubby-like protein (TUBL) [58], a group of membrane-tethered transcription factors involved in the signaling pathway [87] has been found in *Alexandrium*, although this protein has not been fully characterized.

Gene specific transcription factors (TFs) are one of the largest family of proteins in most cells, accounting for ~4% of the genome in yeast or ~8% of the genome in plants and mammals [88]. In contrast, proteins with a DNA binding domain account for only 0.15%–0.3% of the total transcripts in each of two different dinoflagellates, *Lingulodinium* and *Symbiodinium* [41,56]. Furthermore, in both species, roughly two-thirds of the TFs are represented by a single group, the Cold Shock Domain (CSD) containing proteins. The CSD is relatively uncommon in eukaryotes, and importantly, is more often implicated in posttranscriptional than transcriptional regulation [89]. Whether or not the dinoflagellate version of the CSD proteins will be shown to be *bone fide* DNA-binding proteins, and the reason for the preferential expansion of this domain in dinoflagellates, remains to be discovered. However, there is a caveat to assuming that dinoflagellates are bereft of most DNA binding domains based on gene sequence data. Apicomplexans were initially also thought to have a low number of DNA binding proteins, yet further research revealed the expansion of a unique family of transcription factors, ApiAP2, in these organisms [90]. An as yet unknown family of factors modulating transcription may remain to be discovered in the dinoflagellates.

### 2.4. Transcriptional Regulation

Methylation of cytosine in the DNA is a well-studied epigenetic modification that plays an important role in several cellular processes such as retrotransposon silencing, genomic imprinting, X-chromosome inactivation, regulation of gene expression, and maintenance of epigenetic memory [91]. Cytosine methylation occurs at roughly 0.5%–4% of cytosines in dinoflagellates [92,93], and is dynamic as it has been shown to change with varying light conditions [94]. It is thus possible that cytosine methylation may structurally regulate the access of DNA to transcription. In addition to 5-methylcytosine (5-MeC), dinoflagellates possess a number of unusual base modifications such as 5-hydroxymethyluracil (5-HMeU) and N6-methyladenine (N6-MeA) [95]. 5-HMeU is formed in DNA as a product of oxidative attack on the methyl group of thymidine [96], and dinoflagellate DNA contains between 12% and 70% of the thymidine as 5-HMeU [97]. The significance of this modification in dinoflagellate DNA is still unclear.

The posttranslational modification of histones plays an important role in regulating gene expression in other eukaryotes, and deserves re-examination in dinoflagellates because of the recent discovery that conserved sequences for core histones and their regulatory enzymes appear in the transcriptomes [56,71]. It is possible that very low levels of histones are associated with gene regulatory sites, much as the low levels of acetylated histone H3 are associated with initiation of polycistronic transcripts in kinetoplastids [98]. The role of HLPs in regulating gene expression is also unclear, although the sequence-specific DNA binding and their existence in several post-translationally modified forms may indicate an involvement in gene regulatory mechanisms [77]. HLP transcript abundance in dinoflagellates appears to be up-regulated during different phases of cell cycle and in response to nutrient availability, as exemplified by *Pyrocystis lunula* where HLP transcripts peaked during the S-phase [99], and *Alexandrium fundyense* where HLP transcripts were up-regulated during G1 phase [99]. However, unlike the higher eukaryotes whose histone mRNA levels increase during S-phase, no difference in histone mRNAs abundance was found during S-phase in *L. polyedrum* [71]. It will be interesting to examine the newly discovered DVNP [78] to see if transcriptional regulation accompanies DNA synthesis in the dinoflagellates.

Most organisms have evolved an ability to respond to environmental changes, including biotic and abiotic stresses such as changes in light or temperature. The signaling pathways involve receptors that sense and transmit the information to regulatory molecules, and changes in gene expression are a frequently observed cellular response [100]. For example, in *Amphidinium carterae*, Northern blot hybridization revealed that transcript levels of two light harvesting proteins, peridinin chlorophyll *a* protein (PCP) and a major a/c-containing intrinsic light-harvesting proteins (LHC), were, respectively, 86- and 6-fold more abundant under low light conditions than under normal light conditions [94]. Interestingly, this increase in transcript levels coincided with a decrease in DNA cytosine methylation of CpG and CpNpG motifs present near or inside the coding regions of the two genes under low light intensity, although *in vitro* experiments to link DNA demethylation with transcriptional activation were unsuccessful [94]. *Karenia brevis* may also have a transcriptional response to low light, as the abundance of 9.8% of the 4269 unique genes in the microarray differed between day and night [101]. In addition to light, temperature is also an important signal, and has been implicated in the loss of cnidarian-dinoflagellate symbiosis, a phenomenon called coral bleaching. Temperature increases induce oxidative stress in *Symbiodinium bermudense* that result in increased levels of superoxide radicals and hydrogen peroxide [102], and this may be the primary reason for loss of the symbiont [103]. To check the regulation of expression of heat shock protein (*hsp*) genes in *Symbiodinium* residing inside its coral host Acropora millepora, qPCR was used with samples that were subjected to elevated temperatures rapidly or gradually [104]. Dinoflagellate *hsp*70 transcript levels increased from 39% to 57% when temperature increased to 26 °C (moderate) or 29 °C (severe), although when cells were exposed to extreme heat stress *hsp*70 transcript levels decreased by up to 70%. Curiously, *hsp*90 transcript levels always decreased under heat stress and were independent of the speed of the temperature increase [104]. 

Oxidative stress is often able to induce a transcriptional response in organisms. In *L. polyedrum*, metal-induced oxidative stress resulted in sharp increases in the activity of the defense enzyme superoxide dismutase [105], with the increase in activity dependent on the type of metal, its exposure time and concentration [106,107]. This same stress resulted in an increase in the chloroplastic Fe-SOD transcript level, which accounted for the increased enzymatic activity, clearly demonstrating the transcriptional response [108]. Similarly, a microarray of 3500 genes from *P. lunula* revealed that 204 and 37 genes increased in abundance by 2- to 4-fold after treatment with 1 mM sodium nitrite or 0.5 mM paraquat, respectively [109]. The transcriptional response of the heat shock protein genes *hsp*70 and *hsp*90, to elevated temperature, metal and endocrine disrupting chemicals, were tested in the dinoflagellate *Prorocentrum minimum*. RT-PCR results revealed that Hsp70 transcripts increased in response to each of these stresses, while Hsp90 transcript level increased only in response to temperature and metals [110,111]. Lastly, 454 pyrosequencing in the basal dinoflagellate, *Oxyrrhis marina*, revealed 9 and 21 transcripts to be up- and down-regulated by saline stress, respectively [112]. However, it is worth mentioning that transcript levels of only 11 of these 30 genes varied by more than 2-fold, and among these latter, 10 were in the down-regulated class. Clearly, dinoflagellates respond to a variety of stress conditions.

The circadian (daily) clock is an endogenous timer that regulates daily rhythms in organisms from all walks of life [113,114,115,116], and although the clock receives timing cues from light/dark cycles or temperature changes [117,118,119,120], it provides signals distinct from these environmental conditions since rhythms can be maintained under constant conditions. Circadian rhythms presumably make organisms more fit by allowing them to specialize for different tasks at different times of day, and, in many cases, the physiological rhythms regulated by the clock are mediated through changes in gene expression. Indeed, microarray studies showed that the number of circadian mRNAs varied from 5%–20% in *Neurospora*, 10% in *Arabidopsis*, 5%–10% in mice and 30%–65% in the cyanobacteria *Synechococcus elongates* [121,122]. In the dinoflagellate *P. lunula*, 3% of the genes on a microarray were found to exhibit changes in transcript abundance (between 2- and 2.5-fold) [123] while in *K. brevis* 0.7% of the genes varied in both light/dark and constant light (between 2- and 7-fold) [101]. The fluorescence labeling of total RNAs and ^32^P incorporation of ribosomal RNAs in the stationary phase cells of *L. polyedrum* under constant light, followed by subsequent gel electrophoresis of the labeled RNAs, showed circadian rhythmicity with maximum RNA abundance at CT18 [124], the time corresponding to the peak of S-phase in these species [125,126]. However, when *L. polyedrum* cells were treated with Actinomycin D (ActD), a drug that inhibits DNA-dependent RNA synthesis, the bioluminescence and photosynthesis rhythms were unaffected for 30 h or more depending on the dose of the treatment [127]. In contrast to the lack of effect using transcription inhibitors, treatment with translation inhibitor puromycin caused an immediate inhibition of the rhythms [127]. As ActD will also indirectly inhibit protein synthesis when RNA levels have decayed sufficiently, it is possible that the eventual loss of the rhythms following ActD treatment was due to decreasing levels of RNA. Similar tests with high concentrations of other potent inhibitors of RNAP II, such as DRB (5,6-dichloro-1-beta-d-ribofuranosylbenzimidazole) and α-amanitin confirmed no significant effect on growth, luminescence or rhythmicity in *L. polyedrum* cultures [128]. Indeed, all circadian changes of protein levels in *L. polyedrum* have so far proven to be regulated post-transcriptionally [9].

Nutrient availability is also an important environmental cue, and can result in the formation of algal blooms for some dinoflagellates. The nutrients most important for the blooms are nitrogen (N) and phosphorus (P), and thus the transcriptomic response of dinoflagellates to N- and P-deplete and -replete conditions has been of great interest. When *Karenia* grown in N-deplete and -replete conditions were compared, 1102 genes on a microarray chip of 11,000 genes were found to be differentially expressed [129]. Among the up-regulated genes were found type III glutamine synthetases, nitrate/nitrite transporters, and an ammonium transporter, all known to function in the nitrogen uptake and assimilation pathway. The transcriptomic response to P-depletion was not so informative, although 12% of the array showed a different expression profile. However, the activity and transcription levels of alkaline phosphatase were found to be regulated by the availability of the inorganic phosphate source in the dinoflagellates *K*. *brevis* and *A*. *carterae* [130,131]. Interestingly, N and P concentrations and growth stages have a strong impact on the toxin levels produced by *Alexandrium tamarense*, suggesting that expression of genes involved in these pathways may be responsive to nutrients [132]. Microarray experiment with 4298 sequences from *Alexandrium minutum* identified 87 genes that specifically responded to N or P limitation [133], while massively parallel signature sequencing (MPSS) in *A. tamarense* cultures showed only 2 and 12 out of a total of 40,029 signatures were uniquely expressed under N and P starvation, respectively [134].

The strain and growth stage of dinoflagellate cultures can also affect gene expression. In the microarray study of *A. tamarense* discussed above, 489 of the 4298 sequences examined were found to be differentially expressed when exponentially growing and stationary phase cultures were compared, a number even higher than the response induced by nutrient deprivation [133]. Here, proliferating cells showed a greater abundance of translation pathway gene transcripts and a lower abundance of transcripts from genes involved in intracellular signaling [133]. Similar studies in *A. catenella* revealed proliferating cells show over-expression of transcripts from several categories, including transcription and RNA processing, protein synthesis and translational regulation, cell division, transport related, photosynthesis and cellular metabolism [58]. In *Karenia brevis*, five time points representing different growth phases were selected for microarray analysis, and taken together, 21% of the 11,000 features examined had accumulated to different levels in logarithmic compared to stationary phase cells [135]. Interestingly, a comparison of toxic and non-toxic strains of *A. minutum* has indicated a strain specific regulation of gene expression [136]. Using microarray chips with a cut-off value of 1.5 fold difference, 145 and 47 sequences were identified as up-regulated in either toxic or non-toxic strains, respectively. While one of the original goals was to identify toxin-related genes in *Alexandrium*, it is unclear how much reliance can be placed on this line of experiments as many toxin related genes could also have unknown and important metabolic functions in the dinoflagellates and thus be similarly regulate in both strains. This view is supported by the observation that a non-toxic strain of *Heterocapsa circularisquama* transcribes a substantial number of genes thought to be involved in toxin biosynthesis [137]. Lastly, it must be noted that expression of the same gene may be regulated differently in different species. As an example, Rubisco is not subject to transcriptional control in *L. polyedrum* [138] while a pronounced difference is seen in transcript levels over the diurnal cycle in *Prorocentrum donghaiense* [139].

Gene expression in the dinoflagellates can also be influenced by biotic factors, as shown by a massively parallel signature sequencing MPSS comparison of *A. tamarense* grown axenically and in normal cultures [134]. From a total of 40,000 signatures, 307 were differentially expressed in the axenic cultures (39% up-regulated and 61% down-regulated). The association of bacteria with the dinoflagellates seems to affect the methionine-homocysteine cycle and photosynthesis, as these categories were enriched in the differentially expressed genes. However, it is likely that the most important biotic factors will be those related to symbioses. The first indication of symbiosis-specific gene expression in dinoflagellates was obtained from study of *Scrippsiella nutricula* with and without its radiolarian host *Thalassicola nucleata*. It was found that several genes in the dinoflagellate were differentially transcribed depending on symbiotic or free living growth [140]. The dinoflagellate–cnidarian symbiosis, vital for ocean reef ecology, also presents an excellent model for understanding the regulation of gene expression by biotic factors. In this context, a homologue of P-type H^+^-ATPase gene in *Symbiodinium* was shown to be expressed exclusively during the coral symbiosis [141]. Thermal stress, the primary cause of coral bleaching, induced different responses in the host and the symbiont, with the coral expression pattern much more important than the dinoflagellate symbiont [142]. Lastly, copepods (*Calanushel golandicus*, *Acartia clausii*, and *Oithona similis*) were found to induce a species-specific response of toxin production by the dinoflagellate *Alexandrium*, and this was associated with the significant and specific regulation of particular sets of genes, especially those involved in signal transduction, translational and post translational mechanisms [143].

It must be kept in mind that most of the gene regulation studies performed in dinoflagellates are expression-profiling experiments, which indicate mRNA levels and are thus determined by the balance between mRNA synthesis and degradation rates. Indeed, mRNA degradation may play a major role in determining the transcript abundance [144]. So far, only half-lives of transcripts whose protein synthesis is regulated by the clock in the dinoflagellate *L. polyedrum* have been measured [128]. Thus, different mRNA levels obtained during the gene expression studies cannot be unambiguously ascribed to result from transcriptional regulation.

## 3. Splicing and the Spliceosome

Several posttranscriptional modifications in the primary transcripts of eukaryotic cells are necessary to create a mature mRNA that can be efficiently translated, and of these, arguably the most important is the removal of the intervening sequences, or “introns”, that interrupt the coding sequence, or “exons” [145,146,147]. Mammalian genomes are generally intron-rich, while in contrast, dinoflagellate genes contain very few or lack introns completely. For example, all the high copy number genes tested in *L. polyedrum*, such as *pcp*, *lbp* and *lcf*, lack introns [34,35,37]. However, in another bioluminescent dinoflagellate, *P. lunula*, a comparison of genomic and cDNA PCR products of the *lcf C* gene identified a 403 bp intron [38]. The form II Rubisco gene lacks introns in *Prorocentrum minimum* [148], yet contains six introns in *Symbiodinium* [149]. The saxitoxin pathway gene *sxtG* in *Alexandrium* was found to have one intron whose length varied from species to species, ranging from 260 to 750 bp. Sequencing of different sxtG introns showed >90% intraspecies identity and <80% interspecies identity, with no variation observed within a strain [150]. Analyses of hsp90 sequences from the genomic DNA of 17 dinoflagellates reported introns in only three species (97 bp, 134 bp and 289 bp in *Peridinium willei*, *Polarella glacialis* and *Thecadiniium yashimaense*, respectively) [151]*.* A more detailed test, carried out with 31 genes in *A. carterae*, showed that four genes (encoding polyketide synthase, translation initiation factor 3 subunit 8, small nuclear ribonuclear protein and *psbO*) had 6 or more introns, similar to other eukaryotes, another 11 genes had less than 5 introns, and the rest no introns at all [39]. This study also correlated highly expressed genes with a very low intron density and a tandem gene arrangement in the genome [39].

The cellular mechanism that joins exons together by excising the introns is called splicing [146,147]. As expected, splicing must be extremely accurate, as even a single nucleotide frame shift could result in a nonsense mutation or a truncated protein. All introns in the nuclear-encoded pre-mRNAs are delimited by splice sites, which are critical sequences specifying the extremities, and eukaryotic introns are generally bounded by the conserved dinucleotides GU and AG at their 5′ and 3′ ends respectively. Another important sequence, the branch point, is usually located between 18 to 40 nucleotides upstream from the 3′ end of the intron, but except from a mandatory adenine which is ligated to the 5′ end of the intron during the splicing reaction, its sequence is only loosely conserved. Interestingly, the dinoflagellate introns typically lack the usual GU-AG splice sites, as exemplified by the AT-TC intron found in *lcf C* of *P. lunula* [38], the G(C/A)-AG introns in *Symbiodinium* rubisco [149] and the AG-AG intron in the *Alexandrium sxtG* [150]. Some of these novel splice sites have been shown to function in other eukaryotes, such as the introns with GC-AG boundaries described in animal and plant genomes [151]. 

The splice sites in pre-mRNA introns are recognized by base pairing to short RNA molecules (U1, U2, U4, U5 and U6) termed small nuclear RNAs (snRNA), each of which is bound to a complex of proteins to form small nuclear ribonucleoproteins (snRNPs). These five snRNPs, together with numerous non-snRNP proteins, constitute the spliceosome, a dynamic complex that forms and reforms repeatedly to process pre-mRNAs to mature transcripts [152]. Many of the protein components are highly conserved between mammals and dinoflagellates, as evidenced by the observation that autoimmune antibodies recognizing the so-called Smith antigen (Sm protein) present in all five human snRNP complexes were found to recognize four of the *C. cohnii* snRNPs [153]. In addition, the *L. polyedrum* and *Symbiodinium* transcriptomes contain sequences with significant homology to 70% and 85% of the splicing components, respectively [41,56]. A high degree of sequence conservation was also noticed between the dinoflagellate and mammalian U2, U5 and U6 RNAs and, as in higher eukaryotes, the dinoflagellate Sm tends to protect an AUn region in the snRNAs [153]. Furthermore, the snRNAs of dinoflagellates have a modified 5′ trimethylguanosine (TMG) cap, as do snRNAs of other eukaryotes [153]. Intriguingly, the spatial organization of the splicing process in the nucleus also appears similar in dinoflagellates and other eukaryotes. Several phylogenetically different species, including *Prorocentrum micans*, *Alexandrium fundyense*, *Akashiwo sanguinea*, and *Amphidinium carterae* were examined microscopically after immunolabeling with antibodies directed against Sm proteins, DNA and p105-PANA (proliferation associated nuclear antigen) in conjunction with cytochemical staining for RNA, phosphorylated proteins and DNA [154]. These studies revealed a cross-reaction of the anti-Sm with eukaryotic-like perichromosomal granules, structures enriched in splicing factors that are actively involved in splicing, as well as Cajal-like bodies, nuclear regions thought to be involved in the modification and assembly of snRNPs. However, it must be noted that the anti-Sm labeling on Western blots revealed cross-reaction with proteins other than those of the expected molecular weight [154] raising the possibility that atypical Sm antigens may be present in the dinoflagellates.

Despite the paucity of *cis*-splicing events in dinoflagellates, *trans*-splicing is now known to be pervasive [155]. In this, dinoflagellates are similar to the kinetoplastid *Trypanosoma brucei*, where mRNAs were found to contain a consensus sequence of 39 nucleotides (nt) at their 5′ ends. This sequence, termed a spliced leader (SL) sequence [156], is added from a separate SL-donor RNA (an SL RNA) in a process called *trans* splicing to all trypanosome mRNAs [157]. Since this initial discovery, many organisms including cnidarians, ctenophores, flatworms, nematodes, crustaceans, Euglena and now dinoflagellates have also been shown to use SL *trans*-splicing [158,159,160]. The length of the SL exon varies in different species, from 16 nt in *Ciona intestinalis* [161] to 51 nt in *Stylochus zebra* [162], and in dinoflagellates, the SL leader is a 22 nt sequence 5′-DCCGUAGCCAUUUUGGCUCAAG-3′ (D = U, A, or G) [155]. The discovery of the dinoflagellate SL has provided an enormous boost to the study of dinoflagellate molecular biology, in part because full-length sequences of dinoflagellate cDNAs can now be readily retrieved, but more importantly, because dinoflagellate sequences can now be isolated from complex mixtures such as RNA extracted from environmental samples or from organisms in symbiosis [70]. The dinoflagellate SL sequence is derived from SL RNAs of 50–60 nt and contains an Sm binding motif (AUUUUGG) in the exon, unlike all other SL RNAs where this conserved sequence is found in the intron [155]. SL *trans*-splicing is absent in organelle-encoded transcripts, although a unique type of *trans*-splicing was recently found in the mitochondria of diverse dinoflagellates. The mitochondrial *cox3* gene is encoded in two pieces that are transcribed separately then *trans*-spliced to form a complete coding *cox3* mRNA [163]. SL *trans*-splicing is evolutionarily ancient for the dinoflagellates, also being found in the perkinsozoa, *Perkinsus marinus* is basal to the dinoflagellate and apicomplexans lineages, and has nuclear-encoded transcripts with 5 different SL sequences. Three of the SL are 22 nt long and similar to the core dinoflagellates (SL1) while the other two are truncated 21 nt SL with either A or G as the starting nucleotide (SL2) [164]. The function of SL *trans*-splicing is not clear. It is unlikely to be involved in mRNA stability or translation, as there was no difference in translation efficiency or stability between *trans*-spliced and non-*trans*-spliced nematodes mRNAs [160]. It has been proposed that in conjunction with polyadenylation it functions in the production of mature monocistronic transcripts from polycistronic transcripts, and it is still possible that it defines the 5′ end of transcripts even though polycistronic transcription now seems limited [41].

The paucity of introns, as well as the presence of multiple relict sequences related to the SL in the 5′ ends of dinoflagellate genes isolated from genomic DNA, has led to the proposal of a mRNA recycling mechanism whereby mature mRNAs are inserted back into the genome through a recombination process [165]. This hypothesis still requires a more comprehensive enquiry in diverse dinoflagellates, but if true, may shed some light on the origin of the plethora of tandem array genes in dinoflagellates. It is also interesting that alternative splicing, a process by which cells can generate several proteins through permutation and combination of exons from a single pre-mRNA, has been discovered for cyclin transcripts in *Perkinsus marinus* [166]. Alternative splicing may have been lost after divergence from this basal lineage as to date, it has not yet been observed for other dinoflagellates.

## 4. RNA Transport and mRNA Surveillance Pathways

Nuclear pore complexes (NPC) are enormous protein complexes, ranging from 50 MDa in yeast to 125 MDa in mammals, which are present within the nuclear envelope and mediate nucleo-cytoplasmic transport [167,168]. Though small molecules under 40 kDa can passively diffuse through NPC, larger mRNA molecules require a more complex energy-dependent and signal-mediated process [169]. The nuclear export pathway has been well characterized in yeast and higher eukaryotes, but does not appear to be conserved in apicomplexans, as many of the important components are either absent or unrecognizable by homology search algorithms [170]. To date, no description of this pathway has been made in any dinoflagellate, and we have thus analyzed the *L. polyedrum* transcriptome to try and retrieve the components expected for RNA transport. There are three general classes of proteins required, those forming the nuclear pore itself and those soluble in either the nucleus or the cytoplasm. Compared to the components found in other eukaryotes, the most marked difference between the alveolates and other organisms appears to lie in those components used for construction of the pore (Table 1). Apart from the conserved integral membrane proteins termed Nups, thought to anchor the pores in the nuclear membrane, it seems that lower eukaryotes either manage to construct this large molecular complex with far fewer elements than are required in mammals, or alternatively, employ some unique and as yet unidentified constituents. It would evidently be of great interest to examine the structure of the pore using electron microscopy to ascertain if the pore retains the eightfold symmetrical structure normally found in higher eukaryotes. In addition to the NPC, a plethora of nuclear and cytoplasmic *trans* acting factors are also employed to mediate RNA processing and transport in mammals and higher eukaryotes. The nuclear components include factors common to the different types of RNA as well as other specific factors for processing and maturity that facilitate the nucleo-cytoplasmic transport [171], and these appear to be conserved in the dinoflagellates. In contrast, only a third of the mammalian and half of the plant cytoplasmic components involved in nuclear transport are conserved in *L. polyedrum* and other alveolates (Table 1, Supplementary Figure S2).

**Table 1:** Number of components involved in nuclear transport found in the *L. polyedrum* transcriptome. Gene sequences for various Kyoto Encyclopedia of Genes and Genomes (KEGG) pathways were tabulated. The alveolates are represented by *L. polyedrum* (Lp), *Plasmodium falciparum* (Pf) and *Tetrahymena thermophila* (Tt). A cutoff value of e^−25^ was used to assess the presence of components.

		Mammal	Plant	Alveolata			Diatom
				Lp	Pf	Tt	
Nucleus		11	10	6	9	7	8
Central channel	Nuclear basket	4	1	1	0	0	1
	Symmetrical nups	11	9	2	1	4	6
	Central channel	3	3	0	0	0	1
	Spoke complex	5	5	0	0	0	2
	Lumenal ring	3	1	0	0	1	0
	Cytoplasmic tails	8	6	2	2	3	3
Cytoplasm		53	37	17	17	17	24

Eukaryotes also employ a multistep “quality control” or surveillance pathway to selectively degrade the damaged or mutated mRNAs as a protective mechanism against aberrant protein synthesis. This concerted procedure starts with mRNA capping during transcription within the nucleus, and ends in the cytoplasm with the degradation of abnormal mRNAs. There are three main pathways, the first being nonsense-mediated mRNA decay. In mammals, this pathway interprets stop codons found 50 or more nucleotides upstream form the last exon boundary to be premature stop codons, principally because normal stop codon are typically located in the last exon [172,173] and this process uses factors involved in capping or 3′ end processing of the pre-mRNAs as well as a large complex of nuclear factors comprising the exon-junction complex (EJC) as a scaffold [174]. These mRNAs are then degraded to block synthesis of truncated proteins that might act as dominant negative or gain-of-function mutants. Curiously, despite the conservation of many of the components, intron/exon boundaries are not required to fulfill the same role in invertebrates and yeast although the implication of the EJC is not well defined in these systems. Nonsense-mediated decay appears to be operative in dinoflagellates, as many of the generally conserved components are found (Table 2), but the mechanisms used may be more similar to yeasts and insects as dinoflagellate genes have a generally low intron density. The second pathway, termed nonstop-mediated mRNA decay, is used to detect mRNA molecules lacking a stop codon. These transcripts pose a problem in that ribosomes translating into the poly A tail stall and have difficult dissociating from the transcript, thus reducing the number of ribosomes available for general translation [175]. This mechanism requires both a release of the ribosome and a degradation of the mRNA, but the components required for this remain to be fully characterized. Lastly, recognition of stalled ribosomes may also be involved in what is termed no-go mRNA decay [176], where ribosomes stalled during translation, perhaps because of unusual secondary structure elements in the transcript, are also targeted for degradation [174]. In general, dinoflagellates and other alveolates have a very poor conservation of the nuclear factors required for RNA surveillance (27% as compared to mammals) although the conservation of cytoplasmic factors is better (67% as compared to the mammals) (Table 2).

**Table 2:** Number of components involved in mRNA surveillance found in the *L. polyedrum* transcriptome*.* Gene sequences for various KEGG pathways were tabulated. The alveolates are represented by *L. polyedrum* (Lp), *Plasmodium falciparum* (Pf) and *Tetrahymena thermophila* (Tt).A cutoff value of e^−25^ was used to assess the presence of components.

		Mammal	Plant	Alveolata			Diatom
				Lp	Pf	Tt	
Nucleus	Cap binding complex	2	2	0	1	2	1
	EJC	15	11	5	4	4	5
	5′ capping	2	2	0	0	1	2
	Pre-mRNA processing	14	13	4	4	4	8
Cytoplasm	Nonsense mediated decay	12	9	7	6	5	6
	No-go decay	3	3	3	2	2	3

## 5. Conclusions and Perspectives

Considerable progress has been made in the study of dinoflagellate transcription, fuelled in large part by the recent availability of low cost sequencing. We show here that most of the expected players in the transcriptional machinery are found in dinoflagellates, at least with respect to their counterparts among the Alveolata. The exception to this general rule is that the specific transcription factors seem in large part to be reduced in quantity and type in the dinoflagellates. Thus, while general transcription carries on much as expected for the eukaryotes, the specific targeting of genes for transcriptional control may differ as a result of the unusual chromatin organization in this class. Further studies will now be necessary to confirm the biochemical activities of some of the more interesting components identified from the massive influx of sequence information.

## Supplementary Materials

Supplementary File 1

## References

[1] Field C.B., Behrenfeld M.J., Randerson J.T., Falkowski P. (1998). Primary production of the biosphere: Integrating terrestrial and oceanic components. Science.

[2] Muscatine L., McCloskey L.R., Marian R.E. (1981). Estimating the daily contribution of carbon from zooxanthellae to coral animal respiration. Limnol. Oceanogr..

[3] Camacho F.G., Rodriguez J.G., Miron A.S., Garcia M.C., Belarbi E.H., Chisti Y., Grima E.M. (2007). Biotechnological significance of toxic marine dinoflagellates. Biotechnol. Adv..

[4] Schmitter R.E., Njus D., Sulzman F.M., Gooch V.D., Hastings J.W. (1976). Dinoflagellate bioluminescence: A comparative study of *in vitro* components. J. Cell. Physiol..

[5] Hastings J.W., Sweeney B.M. (1958). A persistent diurnal rhythm of luminescence in *Gonyaulax polyedra*. Biol. Bull..

[6] Hastings J.W., Astrachan L., Sweeney B.M. (1961). A persistent daily rhythm in photosynthesis. J. Gen. Physiol..

[7] Sweeney B.M. (1960). The photosynthetic rhythm in single cells of *Gonyaulax polyedra*. Cold Spring Harb. Symp. Quant. Biol..

[8] Roenneberg T., Colfax G.N., Hastings J.W. (1989). A circadian rhythm of population behavior in *Gonyaulax polyedra*. J. Biol. Rhythms.

[9] Hastings J.W. (2007). The Gonyaulax clock at 50: Translational control of circadian expression. Cold Spring Harb. Symp. Quant. Biol..

[10] Fast N.M., Xue L., Bingham S., Keeling P.J. (2002). Re-examining alveolate evolution using multiple protein molecular phylogenies. J. Eukaryot. Microbiol..

[11] Spector D.L., Spector D.L. (1984). Dinoflagellate Nuclei. Dinoflagellates.

[12] Lin S. (2011). Genomic understanding of dinoflagellates. Res. Microbiol..

[13] Wisecaver J.H., Hackett J.D. (2011). Dinoflagellate genome evolution. Annu. Rev. Microbiol..

[14] Hackett J.D., Anderson D.M., Erdner D.L., Bhattacharya D. (2004). Dinoflagellates: A remarkable evolutionary experiment. Am. J. Bot..

[15] Livolant F. (1984). Cholesteric organization of DNA *in vivo* and *in vitro*. Eur. J. Cell Biol..

[16] Livolant F. (1978). Positive and negative birefringence in chromosomes. Chromosoma.

[17] Herzog M., Soyer M.O. (1983). The native structure of dinoflagellate chromosomes and their stabilization by Ca^2+^ and Mg^2+^ cations. Eur. J. Cell Biol..

[18] Sigee D.C. (1983). Structural DNA and genetically active DNA in dinoflagellate chromosomes. Biosystems.

[19] Kornberg R.D. (2007). The molecular basis of eukaryotic transcription. Proc. Natl. Acad. Sci. USA.

[20] Smale S.T., Kadonaga J.T. (2003). The RNA polymerase II core promoter. Annu. Rev. Biochem..

[21] Hahn S. (2004). Structure and mechanism of the RNA polymerase II transcription machinery. Nat. Struct. Mol. Biol..

[22] Carninci P., Sandelin A., Lenhard B., Katayama S., Shimokawa K., Ponjavic J., Semple C.A., Taylor M.S., Engstrom P.G., Frith M.C. (2006). Genome-wide analysis of mammalian promoter architecture and evolution. Nat. Genet..

[23] Everett R.D., Baty D., Chambon P. (1983). The repeated GC-rich motifs upstream from the TATA box are important elements of the SV40 early promoter. Nucleic Acids Res..

[24] Yoshikawa T., Takishita K., Ishida Y., Uchida A. (1997). Molecular cloning and nucleotide sequence analysis of the gene coding for chloroplast-type ferredoxin from the dinoflagellates *Peridinium bipes* and *Alexandrium tamarense*. Fish. Sci..

[25] Wong J.M., Liu F., Bateman E. (1992). Isolation of genomic DNA encoding transcription factor TFIID from *Acanthamoeba castellanii*: Characterization of the promoter. Nucleic Acids Res..

[26] Huang W., Bateman E. (1995). Cloning, expression, and characterization of the TATA-binding protein (TBP) promoter binding factor, a transcription activator of the Acanthamoeba TBP gene. J. Biol. Chem..

[27] Cohen S.M., Knecht D., Lodish H.F., Loomis W.F. (1986). DNA sequences required for expression of a *Dictyostelium* actin gene. EMBO J..

[28] Kimmel A.R., Firtel R.A. (1983). Sequence organization in *Dictyostelium*: Unique structure at the 5′-ends of protein coding genes. Nucleic Acids Res..

[29] Liston D.R., Johnson P.J. (1999). Analysis of a ubiquitous promoter element in a primitive eukaryote: Early evolution of the initiator element. Mol. Cell. Biol..

[30] McAndrew M.B., Read M., Sims P.F., Hyde J.E. (1993). Characterisation of the gene encoding an unusually divergent TATA-binding protein (TBP) from the extremely A+T-rich human malaria parasite *Plasmodium falciparum*. Gene.

[31] Luo H., Gilinger G., Mukherjee D., Bellofatto V. (1999). Transcription initiation at the TATA-less spliced leader RNA gene promoter requires at least two DNA-binding proteins and a tripartite architecture that includes an initiator element. J. Biol. Chem..

[32] Quon D.V., Delgadillo M.G., Johnson P.J. (1996). Transcription in the early diverging eukaryote *Trichomonas vaginalis*: An unusual RNA polymerase II and alpha-amanitin-resistant transcription of protein-coding genes. J. Mol. Evol..

[33] Quon D.V., Delgadillo M.G., Khachi A., Smale S.T., Johnson P.J. (1994). Similarity between a ubiquitous promoter element in an ancient eukaryote and mammalian initiator elements. Proc. Natl. Acad. Sci. USA.

[34] Le Q.H., Markovic P., Hastings J.W., Jovine R.V., Morse D. (1997). Structure and organization of the peridinin-chlorophyll a-binding protein gene in *Gonyaulax polyedra*. Mol. Gen. Genet..

[35] Li L., Hastings J.W. (1998). The structure and organization of the luciferase gene in the photosynthetic dinoflagellate *Gonyaulax polyedra*. Plant Mol. Biol..

[36] Machabee S., Wall L., Morse D. (1994). Expression and genomic organization of a dinoflagellate gene family. Plant Mol. Biol..

[37] Lee D.H., Mittag M., Sczekan S., Morse D., Hastings J.W. (1993). Molecular cloning and genomic organization of a gene for luciferin-binding protein from the dinoflagellate *Gonyaulax polyedra*. J. Biol. Chem..

[38] Okamoto O.K., Liu L., Robertson D.L., Hastings J.W. (2001). Members of a dinoflagellate luciferase gene family differ in synonymous substitution rates. Biochemistry.

[39] Bachvaroff T.R., Place A.R. (2008). From stop to start: Tandem gene arrangement, copy number and *trans*-splicing sites in the dinoflagellate *Amphidinium carterae*. PLoS One.

[40] Jackson A.P. (2007). Tandem gene arrays in *Trypanosoma brucei*: Comparative phylogenomic analysis of duplicate sequence variation. BMC Evol. Biol..

[41] Beauchemin M., Roy S., Daoust P., Dagenais-Bellefeuille S., Bertomeu T., Letourneau L., Lang B.F., Morse D. (2012). Dinoflagellate tandem array gene transcripts are highly conserved and not polycistronic. Proc. Natl. Acad. Sci. USA.

[42] Rizzo P.J. (1979). RNA synthesis in isolated nuclei of the dinoflagellate *Crypthecodinium cohnii*. J. Protozool..

[43] Palenchar J.B., Bellofatto V. (2006). Gene transcription in trypanosomes. Mol. Biochem. Parasitol..

[44] Orphanides G., Lagrange T., Reinberg D. (1996). The general transcription factors of RNA polymerase II. Genes Dev..

[45] Conaway J.W., Bond M.W., Conaway R.C. (1987). An RNA polymerase II transcription system from rat liver. Purification of an essential component. J. Biol. Chem..

[46] Conaway R.C., Conaway J.W. (1989). An RNA polymerase II transcription factor has an associated DNA-dependent ATPase (dATPase) activity strongly stimulated by the TATA region of promoters. Proc. Natl. Acad. Sci. USA.

[47] Conaway J.W., Conaway R.C. (1989). A multisubunit transcription factor essential for accurate initiation by RNA polymerase II. J. Biol. Chem..

[48] Conaway J.W., Reines D., Conaway R.C. (1990). Transcription initiated by RNA polymerase II and purified transcription factors from liver. Cooperative action of transcription factors τ and ε in initial complex formation. J. Biol. Chem..

[49] Sumimoto H., Ohkuma Y., Yamamoto T., Horikoshi M., Roeder R.G. (1990). Factors involved in specific transcription by mammalian RNA polymerase II: Identification of general transcription factor TFIIG. Proc. Natl. Acad. Sci. USA.

[50] Poon D., Bai Y., Campbell A.M., Bjorklund S., Kim Y.J., Zhou S., Kornberg R.D., Weil P.A. (1995). Identification and characterization of a TFIID-like multiprotein complex from *Saccharomyces cerevisiae*. Proc. Natl. Acad. Sci. USA.

[51] Sanders S.L., Weil P.A. (2000). Identification of two novel TAF subunits of the yeast *Saccharomyces cerevisiae* TFIID complex. J. Biol. Chem..

[52] Chatterjee S., Struhl K. (1995). Connecting a promoter-bound protein to TBP bypasses the need for a transcriptional activation domain. Nature.

[53] Chong J.A., Moran M.M., Teichmann M., Kaczmarek J.S., Roeder R., Clapham D.E. (2005). TATA-binding protein (TBP)-like factor (TLF) is a functional regulator of transcription: Reciprocal regulation of the neurofibromatosis type 1 and c-fos genes by TLF/TRF2 and TBP. Mol. Cell. Biol..

[54] Holmes M.C., Tjian R. (2000). Promoter-selective properties of the TBP-related factor TRF1. Science.

[55] Guillebault D., Sasorith S., Derelle E., Wurtz J.M., Lozano J.C., Bingham S., Tora L., Moreau H. (2002). A new class of transcription initiation factors, intermediate between TATA box-binding proteins (TBPs) and TBP-like factors (TLFs), is present in the marine unicellular organism, the dinoflagellate *Crypthecodinium cohnii*. J. Biol. Chem..

[56] Bayer T., Aranda M., Sunagawa S., Yum L.K., Desalvo M.K., Lindquist E., Coffroth M.A., Voolstra C.R., Medina M. (2012). Symbiodinium transcriptomes: Genome insights into the dinoflagellate symbionts of reef-building corals. PLoS One.

[57] Tamura K., Peterson D., Peterson N., Stecher G., Nei M., Kumar S. (2011). MEGA5: Molecular evolutionary genetics analysis using maximum likelihood, evolutionary distance, and maximum parsimony methods. Mol. Biol. Evol..

[58] Toulza E., Shin M.S., Blanc G., Audic S., Laabir M., Collos Y., Claverie J.M., Grzebyk D. (2010). Gene expression in proliferating cells of the dinoflagellate *Alexandrium catenella* (Dinophyceae). Appl. Environ. Microbiol..

[59] Qiu X.B., Lin Y.L., Thome K.C., Pian P., Schlegel B.P., Weremowicz S., Parvin J.D., Dutta A. (1998). An eukaryotic RuvB-like protein (RUVBL1) essential for growth. J. Biol. Chem..

[60] Luger K., Mader A.W., Richmond R.K., Sargent D.F., Richmond T.J. (1997). Crystal structure of the nucleosome core particle at 2.8 A resolution. Nature.

[61] Eickbush T.H., Moudrianakis E.N. (1978). The histone core complex: An octamer assembled by two sets of protein-protein interactions. Biochemistry.

[62] Kasinsky H.E., Lewis J.D., Dacks J.B., Ausio J. (2001). Origin of H1 linker histones. FASEB J..

[63] Rizzo P.J. (2003). Those amazing dinoflagellate chromosomes. Cell Res..

[64] Rizzo P.J. (1981). Comparative aspects of basic chromatin proteins in dinoflagellates. Biosystems.

[65] Vernet G., Sala-Rovira M., Maeder M., Jacques F., Herzog M. (1990). Basic nuclear proteins of the histone-less eukaryote *Crypthecodinium cohnii* (Pyrrhophyta): Two-dimensional electrophoresis and DNA-binding properties. Biochim. Biophys. Acta.

[66] Bodansky S., Mintz L.B., Holmes D.S. (1979). The mesokaryote *Gyrodinium cohnii* lacks nucleosomes. Biochem. Biophys. Res. Commun..

[67] Rizzo P.J., Nooden L.D. (1972). Chromosomal proteins in the dinoflagellate alga *Gyrodinium cohnii*. Science.

[68] Livolant F. (1984). Cholesteric organization of DNA in the stallion sperm head. Tissue Cell.

[69] Balhorn R. (2007). The protamine family of sperm nuclear proteins. Genome Biol..

[70] Lin S., Zhang H., Zhuang Y., Tran B., Gill J. (2010). Spliced leader-based metatranscriptomic analyses lead to recognition of hidden genomic features in dinoflagellates. Proc. Natl. Acad. Sci. USA.

[71] Roy S., Morse D. (2012). A full suite of histone and histone modifying genes are transcribed in the dinoflagellate *Lingulodinium*. PLoS One.

[72] Rizzo P.J., Jones M., Ray S.M. (1982). Isolation and properties of isolated nuclei from the Florida red tide dinoflagellate *Gymnodinium breve* (Davis). J. Protozool..

[73] Kellenberger E., Arnold-Schulz-Gahmen B. (1992). Chromatins of low-protein content: Special features of their compaction and condensation. FEMS Microbiol. Lett..

[74] Holck A., Lossius I., Aasland R., Haarr L., Kleppe K. (1987). DNA- and RNA-binding proteins of chromatin from *Escherichia coli*. Biochim. Biophys. Acta.

[75] Wong J.T., New D.C., Wong J.C., Hung V.K. (2003). Histone-like proteins of the dinoflagellate *Crypthecodinium cohnii* have homologies to bacterial DNA-binding proteins. Eukaryot. Cell.

[76] Sala-Rovira M., Geraud M.L., Caput D., Jacques F., Soyer-Gobillard M.O., Vernet G., Herzog M. (1991). Molecular cloning and immunolocalization of two variants of the major basic nuclear protein (HCc) from the histone-less eukaryote *Crypthecodinium cohnii* (Pyrrhophyta). Chromosoma.

[77] Chudnovsky Y., Li J.F., Rizzo P.J., Hastings J.W., Fagan T. (2002). Cloning, expression, and characterization of a histone-like protein from the marine dinoflagellate *Lingulodinium polyedrum*. J. Phycol..

[78] Gornik S.G., Ford K.L., Mulhern T.D., Bacic A., McFadden G.I., Waller R.F. (2012). Loss of nucleosomal DNA condensation coincides with appearance of a novel nuclear protein in dinoflagellates. Curr. Biol..

[79] Azevedo C. (1989). Fine structure of *Perkinsus atlanticus* n. sp. (Apicomplexa, Perkinsea) parasite of the clam *Ruditapes decussatus* from Portugal. J. Parasitol..

[80] Jaeckisch N., Yang I., Wohlrab S., Glockner G., Kroymann J., Vogel H., Cembella A., John U. (2011). Comparative genomic and transcriptomic characterization of the toxigenic marine dinoflagellate *Alexandrium ostenfeldii*. PLoS One.

[81] Minguez A., Franca S., Moreno Diaz de la Espina S. (1994). Dinoflagellates have a eukaryotic nuclear matrix with lamin-like proteins and topoisomerase II. J. Cell Sci..

[82] Dechat T., Pfleghaar K., Sengupta K., Shimi T., Shumaker D.K., Solimando L., Goldman R.D. (2008). Nuclear lamins: Major factors in the structural organization and function of the nucleus and chromatin. Genes Dev..

[83] Dechat T., Adam S.A., Goldman R.D. (2009). Nuclear lamins and chromatin: When structure meets function. Adv. Enzym. Regul..

[84] Dechat T., Adam S.A., Taimen P., Shimi T., Goldman R.D. (2010). Nuclear lamins. Cold Spring Harb. Perspect. Biol..

[85] Bhaud Y., Geraud M.L., Ausseil J., Soyer-Gobillard M.O., Moreau H. (1999). Cyclic expression of a nuclear protein in a dinoflagellate. J. Eukaryot. Microbiol..

[86] Guillebault D., Derelle E., Bhaud Y., Moreau H. (2001). Role of nuclear WW domains and proline-rich proteins in dinoflagellate transcription. Protist.

[87] Boggon T.J., Shan W.S., Santagata S., Myers S.C., Shapiro L. (1999). Implication of tubby proteins as transcription factors by structure-based functional analysis. Science.

[88] Babu M.M., Luscombe N.M., Aravind L., Gerstein M., Teichmann S.A. (2004). Structure and evolution of transcriptional regulatory networks. Curr. Opin. Struct. Biol..

[89] Sommerville J. (1999). Activities of cold-shock domain proteins in translation control. Bioessays.

[90] Balaji S., Babu M.M., Iyer L.M., Aravind L. (2005). Discovery of the principal specific transcription factors of Apicomplexa and their implication for the evolution of the AP2-integrase DNA binding domains. Nucleic Acids Res..

[91] Bird A. (2002). DNA methylation patterns and epigenetic memory. Genes Dev..

[92] Blank R.J., Huss V.A.R., Kersten W. (1988). Base composition of DNA from symbiotic dinoflagellates: A tool for phylogenetic classification. Arch. Microbiol..

[93] Steele R.E., Rae P.M. (1980). Ordered distribution of modified bases in the DNA of a dinoflagellate. Nucleic Acids Res..

[94] Ten Lohuis M.R., Miller D.J. (1998). Light-regulated transcription of genes encoding peridinin chlorophyll a proteins and the major intrinsic light-harvesting complex proteins in the dinoflagellate amphidinium carterae hulburt (Dinophycae). Changes In cytosine methylation accompany photoadaptation. Plant Physiol..

[95] Rae P.M., Steele R.E. (1978). Modified bases in the DNAs of unicellular eukaryotes: An examination of distributions and possible roles, with emphasis on hydroxymethyluracil in dinoflagellates. Biosystems.

[96] Teebor G.W., Frenkel K., Goldstein M.S. (1984). Ionizing radiation and tritium transmutation both cause formation of 5-hydroxymethyl-2′-deoxyuridine in cellular DNA. Proc. Natl. Acad. Sci. USA.

[97] Rae P.M. (1976). Hydroxymethyluracil in eukaryote DNA: A natural feature of the pyrrophyta (dinoflagellates). Science.

[98] Thomas S., Green A., Sturm N.R., Campbell D.A., Myler P.J. (2009). Histone acetylations mark origins of polycistronic transcription in *Leishmania major*. BMC Genomics.

[99] Wong J.T., Kwok A.C. (2005). Proliferation of dinoflagellates: Blooming or bleaching. Bioessays.

[100] Gyula P., Schafer E., Nagy F. (2003). Light perception and signalling in higher plants. Curr. Opin. Plant Biol..

[101] Van Dolah F.M., Lidie K.B., More J.S., Brunelle S.A., Ryan J.C., Monroe E.A., Haynes B.L. (2007). Microarray analysis of diurnal- and circadian-regulated genes in the Florida red-tide dinoflagellate *Karenia brevis* (Dinophyceae). J. Phycol..

[102] Lesser M.P. (1996). Elevated temperatures and ultraviolet radiation cause oxidative stress and inhibit photosynthesis in symbiotic dinoflagellates. Limnol. Oceanogr..

[103] Lesser M.P. (1997). Oxidative stress causes coral bleaching during exposure to elevated temperatures. Coral Reefs.

[104] Rosic N.N., Pernice M., Dove S., Dunn S., Hoegh-Guldberg O. (2010). Gene expression profiles of cytosolic heat shock proteins Hsp70 and Hsp90 from symbiotic dinoflagellates in response to thermal stress: Possible implications for coral bleaching. Cell Stress Chaperones.

[105] Walsh C.T., Garneau-Tsodikova S., Gatto G.J. (2005). Protein posttranslational modifications: The chemistry of proteome diversifications. Angew. Chem. Int. Ed. Engl..

[106] Okamoto O.K., Asano C.S., Aidar E., Colepicolo P. (1996). Of cadmium on growth and superoxide dismutase activity of this species of dinoflagellate. The marine microalga *Tetraselmis gracilis*. J. Phycol..

[107] Okamoto O.K., Colepicolo P. (1998). Response of superoxide dismutase to pollutant metal stress in the marine dinoflagellate *Gonyaulax polyedra*. Comp. Biochem. Physiol. C Pharmacol. Toxicol. Endocrinol..

[108] Okamoto O.K., Robertson D.L., Fagan T.F., Hastings J.W., Colepicolo P. (2001). Different regulatory mechanisms modulate the expression of a dinoflagellate iron-superoxide dismutase. J. Biol. Chem..

[109] Okamoto O.K., Hastings J.W. (2003). Genome-wide analysis of redox-regulated genes in a dinoflagellate. Gene.

[110] Guo R., Ebenezer V., Ki J.S. (2012). Transcriptional responses of heat shock protein 70 (Hsp70) to thermal, bisphenol A, and copper stresses in the dinoflagellate *Prorocentrum minimu*. Chemosphere.

[111] Guo R., Ki J.S. (2012). Differential transcription of heat shock protein 90 (HSP90) in the dinoflagellate *Prorocentrum minimum* by copper and endocrine-disrupting chemicals. Ecotoxicology.

[112] Lowe C.D., Mello L.V., Samatar N., Martin L.E., Montagnes D.J., Watts P.C. (2011). The transcriptome of the novel dinoflagellate *Oxyrrhis marina* (Alveolata: Dinophyceae): Response to salinity examined by 454 sequencing. BMC Genomics.

[113] Kondo T., Ishiura M. (2000). The circadian clock of cyanobacteria. Bioessays.

[114] McClung C.R. (2006). Plant circadian rhythms. Plant Cell.

[115] Loros J.J., Dunlap J.C. (2001). Genetic and molecular analysis of circadian rhythms in *Neurospora*. Annu. Rev. Physiol..

[116] Rivkees S.A. (2007). The development of circadian rhythms: From animals to humans. Sleep Med. Clin..

[117] Roenneberg T., Rehman J. (1996). Nitrate, a nonphotic signal for the circadian system. FASEB J..

[118] Roenneberg T., Merrow M. (2007). Entrainment of the human circadian clock. Cold Spring Harb. Symp. Quant. Biol..

[119] Merrow M., Roenneberg T. (2007). Circadian entrainment of *Neurospora crassa*. Cold Spring Harb. Symp. Quant. Biol..

[120] Roenneberg T., Kumar C.J., Merrow M. (2007). The human circadian clock entrains to sun time. Curr. Biol..

[121] Woelfle M.A., Johnson C.H. (2006). No promoter left behind: Global circadian gene expression in cyanobacteria. J. Biol. Rhythms.

[122] Ito H., Mutsuda M., Murayama Y., Tomita J., Hosokawa N., Terauchi K., Sugita C., Sugita M., Kondo T., Iwasaki H. (2009). Cyanobacterial daily life with Kai-based circadian and diurnal genome-wide transcriptional control in *Synechococcus elongatus*. Proc. Natl. Acad. Sci. USA.

[123] Okamoto O.K., Hastings J.W. (2003). Novel dinoflagellate clock-related genes identified through microarray analysis. J. Phycol..

[124] Walz B., Walz A., Sweeney B.M. (1983). A circadian rhythm in RNA in the dinoflagellate, *Gonyaulax polyedra*. J. Comp. Physiol..

[125] Dagenais-Bellefeuille S., Bertomeu T., Morse D. (2008). S-phase and M-phase timing are under independent circadian control in the dinoflagellate *Lingulodinium*. J. Biol. Rhythms.

[126] Bertomeu T., Rivoal J., Morse D. (2007). A dinoflagellate CDK5-like cyclin-dependent kinase. Biol. Cell.

[127] Karakashian M.W., Hastings J.W. (1962). The inhibition of a biological clock by actinomycin D. Proc. Natl. Acad. Sci. USA.

[128] Rossini C., Taylor W., Fagan T., Hastings J.W. (2003). Lifetimes of mRNAs for clock-regulated proteins in a dinoflagellate. Chronobiol. Int..

[129] Morey J.S., Monroe E.A., Kinney A.L., Beal M., Johnson J.G., Hitchcock G.L., van Dolah F.M. (2011). Transcriptomic response of the red tide dinoflagellate, *Karenia brevis*, to nitrogen and phosphorus depletion and addition. BMC Genomics.

[130] Lin X., Zhang H., Huang B., Lin S. (2011). Alkaline phosphatase gene sequence and transcriptional regulation by phosphate limitation in *Amphidinium carterae* (dinophyceae). J. Phycol..

[131] Lin X., Zhang H., Huang B., Lin S. (2012). Alkaline phosphatase gene sequence characteristics and transcriptional regulation by phosphate limitation in *Karenia brevis* (Dinophyceae). Harmful Algae.

[132] Lee T.C., Kwok O.T., Ho K.C., Lee F.W. (2012). Effects of different nitrate and phosphate concentrations on the growth and toxin production of an *Alexandrium tamarense* strain collected from Drake Passage. Mar. Environ. Res..

[133] Yang I., Beszteri S., Tillmann U., Cembella A., John U. (2011). Growth- and nutrient-dependent gene expression in the toxigenic marine dinoflagellate *Alexandrium minutum*. Harmful Algae.

[134] Moustafa A., Evans A.N., Kulis D.M., Hackett J.D., Erdner D.L., Anderson D.M., Bhattacharya D. (2010). Transcriptome profiling of a toxic dinoflagellate reveals a gene-rich protist and a potential impact on gene expression due to bacterial presence. PLoS One.

[135] Johnson J.G., Morey J.S., Neely M.G., Ryan J.C., van Dolah F.M. (2012). Transcriptome remodeling associated with chronological aging in the dinoflagellate, *Karenia brevis*. Mar. Genomics.

[136] Yang I., John U., Beszteri S., Glockner G., Krock B., Goesmann A., Cembella A.D. (2010). Comparative gene expression in toxic *versus* non-toxic strains of the marine dinoflagellate *Alexandrium minutum*. BMC Genomics.

[137] Salcedo T., Upadhyay R.J., Nagasaki K., Bhattacharya D. (2012). Dozens of toxin-related genes are expressed in a nontoxic strain of the dinoflagellate *Heterocapsa circularisquama*. Mol. Biol. Evol..

[138] Nassoury N., Cappadocia M., Morse D. (2003). Plastid ultrastructure defines the protein import pathway in dinoflagellates. J. Cell Sci..

[139] Shi X., Zhang H., Lin S. (2013). Tandem repeats, high copy number and remarkable diel expression rhythm of form II RuBisCO in *Prorocentrum donghaiense* (dinophyceae). PLoS One.

[140] Gast R.J., Beaudoin D.J., Caron D.A. (2003). Isolation of symbiotically expressed genes from the dinoflagellate symbiont of the solitary radiolarian *Thalassicolla nucleata*. Biol. Bull..

[141] Bertucci A., Tambutte E., Tambutte S., Allemand D., Zoccola D. (2010). Symbiosis-dependent gene expression in coral-dinoflagellate association: Cloning and characterization of a P-type H^+^-ATPase gene. Proc. Biol. Sci..

[142] Leggat W., Seneca F., Wasmund K., Ukani L., Yellowlees D., Ainsworth T.D. (2011). Differential responses of the coral host and their algal symbiont to thermal stress. PLoS One.

[143] Wohlrab S., Iversen M.H., John U. (2010). A molecular and co-evolutionary context for grazer induced toxin production in *Alexandrium tamarense*. PLoS One.

[144] Yang E., van Nimwegen E., Zavolan M., Rajewsky N., Schroeder M., Magnasco M., Darnell J.E. (2003). Decay rates of human mRNAs: Correlation with functional characteristics and sequence attributes. Genome Res..

[145] Kinniburgh A.J., Mertz J.E., Ross J. (1978). The precursor of mouse beta-globin messenger RNA contains two intervening RNA sequences. Cell.

[146] Chow L.T., Gelinas R.E., Broker T.R., Roberts R.J. (1977). An amazing sequence arrangement at the 5′ ends of adenovirus 2 messenger RNA. Cell.

[147] Berget S.M., Moore C., Sharp P.A. (1977). Spliced segments at the 5′ terminus of adenovirus 2 late mRNA. Proc. Natl. Acad. Sci. USA.

[148] Zhang H., Lin S. (2003). Complex gene structure of the form II Rubisco in the dinoflagellate *Prorocentrum minimum* (Dinophyceae). J. Phycol..

[149] Rowan R., Whitney S.M., Fowler A., Yellowlees D. (1996). Rubisco in marine symbiotic dinoflagellates: Form II enzymes in eukaryotic oxygenic phototrophs encoded by a nuclear multigene family. Plant Cell.

[150] Orr R.J., Stuken A., Murray S.A., Jakobsen K.S. (2013). Evolutionary acquisition and loss of saxitoxin biosynthesis in dinoflagellates: The second “core” gene, sxtG. Appl. Environ. Microbiol..

[151] Kitamura-Abe S., Itoh H., Washio T., Tsutsumi A., Tomita M. (2004). Characterization of the splice sites in GT-AG and GC-AG introns in higher eukaryotes using full-length cDNAs. J. Bioinform. Comput. Biol..

[152] Nilsen T.W. (2002). The spliceosome: No assembly required?. Mol. Cell.

[153] Reddy R., Spector D., Henning D., Liu M.H., Busch H. (1983). Isolation and partial characterization of dinoflagellate U1–U6 small RNAs homologous to rat U small nuclear RNAs. J. Biol. Chem..

[154] Alverca E., Franca S., Diaz de la Espina S.M. (2006). Topology of splicing and snRNP biogenesis in dinoflagellate nuclei. Biol. Cell.

[155] Zhang H., Hou Y., Miranda L., Campbell D.A., Sturm N.R., Gaasterland T., Lin S. (2007). Spliced leader RNA *trans*-splicing in dinoflagellates. Proc. Natl. Acad. Sci. USA.

[156] Boothroyd J.C., Cross G.A. (1982). Transcripts coding for variant surface glycoproteins of *Trypanosoma brucei* have a short, identical exon at their 5′ end. Gene.

[157] Agabian N. (1990). *Trans* splicing of nuclear pre-mRNAs. Cell.

[158] Douris V., Telford M.J., Averof M. (2010). Evidence for multiple independent origins of *trans*-splicing in Metazoa. Mol. Biol. Evol..

[159] Hastings K.E. (2005). SL *trans*-splicing: Easy come or easy go?. Trends Genet..

[160] Lasda E.L., Blumenthal T. (2011). *Trans*-splicing. Wiley Interdiscip. Rev. RNA.

[161] Vandenberghe A.E., Meedel T.H., Hastings K.E. (2001). mRNA 5′-leader *trans*-splicing in the chordates. Genes Dev..

[162] Davis R.E. (1997). Surprising diversity and distribution of spliced leader RNAs in flatworms. Mol. Biochem. Parasitol..

[163] Jackson C.J., Waller R.F. (2013). A widespread and unusual RNA *trans*-splicing type in dinoflagellate mitochondria. PLoS One.

[164] Hearne J.L., Pitula J.S. (2011). Identification of two spliced leader RNA transcripts from *Perkinsus marinus*. J. Eukaryot. Microbiol..

[165] Slamovits C.H., Keeling P.J. (2008). Widespread recycling of processed cDNAs in dinoflagellates. Curr. Biol..

[166] Zhang H., Dungan C.F., Lin S. (2011). Introns, alternative splicing, spliced leader *trans*-splicing and differential expression of pcna and cyclin in *Perkinsus marinu*. Protist.

[167] Suntharalingam M., Wente S.R. (2003). Peering through the pore: Nuclear pore complex structure, assembly, and function. Dev. Cell.

[168] Vasu S.K., Forbes D.J. (2001). Nuclear pores and nuclear assembly. Curr. Opin. Cell Biol..

[169] Fried H., Kutay U. (2003). Nucleocytoplasmic transport: Taking an inventory. Cell. Mol. Life Sci..

[170] Frankel M.B., Knoll L.J. (2009). The ins and outs of nuclear trafficking: Unusual aspects in apicomplexan parasites. DNA Cell Biol..

[171] Kohler A., Hurt E. (2007). Exporting RNA from the nucleus to the cytoplasm. Nat. Rev. Mol. Cell Biol..

[172] Zhang J., Sun X., Qian Y., LaDuca J.P., Maquat L.E. (1998). At least one intron is required for the nonsense-mediated decay of triosephosphate isomerase mRNA: A possible link between nuclear splicing and cytoplasmic translation. Mol. Cell. Biol..

[173] Zhang J., Sun X., Qian Y., Maquat L.E. (1998). Intron function in the nonsense-mediated decay of beta-globin mRNA: Indications that pre-mRNA splicing in the nucleus can influence mRNA translation in the cytoplasm. RNA.

[174] Isken O., Maquat L.E. (2007). Quality control of eukaryotic mRNA: Safeguarding cells from abnormal mRNA function. Genes Dev..

[175] Ito-Harashima S., Kuroha K., Tatematsu T., Inada T. (2007). Translation of the poly(A) tail plays crucial roles in nonstop mRNA surveillance via translation repression and protein destabilization by proteasome in yeast. Genes Dev..

[176] Wu S., Wang W., Kong X., Congdon L.M., Yokomori K., Kirschner M.W., Rice J.C. (2010). Dynamic regulation of the PR-Set7 histone methyltransferase is required for normal cell cycle progression. Genes Dev..

