# Supplementary material for: Transcription and Maturation of mRNA in Dinoflagellates
Source: Microorganisms. 2013 Nov 1;1(1):71–99. doi: 10.3390/microorganisms1010071 (PMC5029490; doi:10.3390/microorganisms1010071)

Supplementary Information

Figure S1. A matrix showing the number of sequence identities between TBP and TBP-like (TLF) sequences from a range of different species.

		1	2	3	4	5	6	7	8	9	10	11	12	13	14	15	16	17	18	19	20	21	22	23	24	25	26	27	28	29	30	31	32	33	34	35	36	37
**1**	Homo_sapiens		308	278	271	297	194	187	142	157	155	153	146	142	151	159	152	95	75	68	73	101	65	74	75	76	97	70	58	73	98	61	73	78	49	70	108	108
**2**	Mus_musculus	308		278	271	294	188	187	142	156	154	152	144	141	151	158	152	95	75	68	73	101	65	74	75	74	98	70	58	73	98	62	73	79	49	70	109	108
**3**	Xenopus_laevis	278	278		264	279	187	186	142	156	154	152	144	142	151	154	150	96	75	65	73	101	63	75	74	74	97	72	57	73	101	64	72	78	49	67	108	108
**4**	Danio_rerio	271	271	264		272	186	187	141	154	153	153	146	141	151	156	150	95	75	68	73	101	64	73	76	77	99	71	57	70	98	62	72	78	49	70	108	108
**5**	Gallus_gallus	297	294	279	272		188	187	142	157	155	154	146	142	151	160	152	95	75	68	73	101	65	75	74	74	96	70	58	72	98	62	73	78	49	70	108	108
**6**	Drosophila_melanogaster	194	188	187	186	188		181	141	149	150	149	144	141	153	157	148	96	76	62	73	98	57	78	79	71	94	69	52	69	95	68	69	69	47	65	111	106
**7**	Strongylocentrotus_purpuratus	187	187	186	187	187	181		141	150	150	150	142	141	153	149	148	94	74	60	69	98	58	75	72	76	97	68	59	67	91	65	67	75	47	67	110	105
**8**	Arabidopsis_thaliana	142	142	142	141	142	141	141		166	166	162	137	136	138	140	142	86	70	52	69	93	52	71	74	57	89	64	53	61	91	54	61	70	49	62	102	96
**9**	Glycine_max	157	156	156	154	157	149	150	166		191	189	147	145	158	156	158	93	77	56	74	104	55	77	79	57	96	70	54	64	98	55	69	76	51	64	107	104
**10**	Zea_mays	155	154	154	153	155	150	150	166	191		189	149	146	158	158	161	95	77	57	74	103	55	77	79	58	95	70	54	66	96	56	69	73	52	67	108	106
**11**	Oryza_sativa	153	152	152	153	154	149	150	162	189	189		150	145	154	159	158	95	76	56	74	104	55	76	80	58	95	71	54	61	96	59	67	75	51	65	107	104
**12**	Chlamydomonas_reinhardtii	146	144	144	146	146	144	142	137	147	149	150		195	141	147	147	95	82	58	73	98	59	73	74	60	98	65	57	63	98	58	59	77	49	61	107	100
**13**	Volvox carteri	142	141	142	141	142	141	141	136	145	146	145	195		139	141	142	95	82	58	73	99	59	72	74	58	96	59	56	60	96	53	57	76	49	61	108	100
**14**	Chlamydomonas reinhardtii	151	151	151	151	151	153	153	138	158	158	154	141	139		169	165	95	77	62	72	109	57	87	79	59	96	73	58	74	98	56	69	75	51	65	106	105
**15**	Neurospora crassa	159	158	154	156	160	157	149	140	156	158	159	147	141	169		176	96	80	64	75	103	61	82	79	59	96	70	60	67	97	62	72	73	53	67	103	102
**16**	Zea mays	152	152	150	150	152	148	148	142	158	161	158	147	142	165	176		95	79	59	74	102	57	79	75	55	94	68	54	67	93	55	67	71	53	64	103	102
**17**	Cryptosporidium_muris	95	95	96	95	95	96	94	86	93	95	95	95	95	95	96	95		64	56	52	162	47	103	100	57	94	63	50	62	93	51	63	80	51	68	79	77
**18**	Tetrahymena_thermophila	75	75	75	75	75	76	74	70	77	77	76	82	82	77	80	79	64		41	103	65	57	56	51	43	62	46	38	41	62	42	45	50	37	40	71	65
**19**	Toxoplasma_gondii	68	68	65	68	68	62	60	52	56	57	56	58	58	62	64	59	56	41		40	55	72	51	53	44	58	47	37	59	56	44	48	53	40	61	50	49
**20**	Ichthyophthirius_multifiliis	73	73	73	73	73	73	69	69	74	74	74	73	73	72	75	74	52	103	40		55	39	48	49	38	61	44	32	42	61	34	39	52	35	41	72	66
**21**	Cryptosporidium_parvum	101	101	101	101	101	98	98	93	104	103	104	98	99	109	103	102	162	65	55	55		48	105	105	58	103	60	47	66	103	49	64	81	50	70	80	79
**22**	Babesia_bovis	65	65	63	64	65	57	58	52	55	55	55	59	59	57	61	57	47	57	72	39	48		53	53	35	53	40	34	39	53	36	44	51	38	47	47	51
**23**	Plasmodium_vivax	74	74	75	73	75	78	75	71	77	77	76	73	72	87	82	79	103	56	51	48	105	53		154	47	86	61	44	62	80	52	61	80	48	57	67	64
**24**	Plasmodium_falciparum	75	75	74	76	74	79	72	74	79	79	80	74	74	79	79	75	100	51	53	49	105	53	154		48	82	55	40	59	80	46	56	81	45	62	69	63
**25**	Lingulodinium polyedrum TLF1	76	74	74	77	74	71	76	57	57	58	58	60	58	59	59	55	57	43	44	38	58	35	47	48		71	81	65	88	65	88	97	56	66	78	54	52
**26**	Perkinsus marinus TLF1	97	98	97	99	96	94	97	89	96	95	95	98	96	96	96	94	94	62	58	61	103	53	86	82	71		62	61	83	214	71	71	107	56	78	84	79
**27**	Symbiodinium kb8 TLF1	70	70	72	71	70	69	68	64	70	70	71	65	59	73	70	68	63	46	47	44	60	40	61	55	81	62		62	136	64	79	139	59	99	110	60	61
**28**	Symbiodinium mf105 TLF1	58	58	57	57	58	52	59	53	54	54	54	57	56	58	60	54	50	38	37	32	47	34	44	40	65	61	62		73	64	97	70	54	50	61	45	46
**29**	Lingulodinium polyedrum TLF2	73	73	73	70	72	69	67	61	64	66	61	63	60	74	67	67	62	41	59	42	66	39	62	59	88	83	136	73		82	77	134	71	92	114	62	60
**30**	Perkinsus marinus TLF2	98	98	101	98	98	95	91	91	98	96	96	98	96	98	97	93	93	62	56	61	103	53	80	80	65	214	64	64	82		73	73	113	56	76	84	79
**31**	Symbiodinium kb8 TLF2	61	62	64	62	62	68	65	54	55	56	59	58	53	56	62	55	51	42	44	34	49	36	52	46	88	71	79	97	77	73		81	59	56	61	47	49
**32**	Symbiodinium mf105 TLF2	73	73	72	72	73	69	67	61	69	69	67	59	57	69	72	67	63	45	48	39	64	44	61	56	97	71	139	70	134	73	81		65	143	108	56	60
**33**	Perkinsus marinus TLF3	78	79	78	78	78	69	75	70	76	73	75	77	76	75	73	71	80	50	53	52	81	51	80	81	56	107	59	54	71	113	59	65		49	69	68	61
**34**	Symbiodinium mf105 TLF3	49	49	49	49	49	47	47	49	51	52	51	49	49	51	53	53	51	37	40	35	50	38	48	45	66	56	99	50	92	56	56	143	49		84	47	47
**35**	Crypthecodinium cohnii TLF	70	70	67	70	70	65	67	62	64	67	65	61	61	65	67	64	68	40	61	41	70	47	57	62	78	78	110	61	114	76	61	108	69	84		63	61
**36**	Phaeodactylum tricornutum	108	109	108	108	108	111	110	102	107	108	107	107	108	106	103	103	79	71	50	72	80	47	67	69	54	84	60	45	62	84	47	56	68	47	63		125
**37**	Thalassiosira pseudonana	108	108	108	108	108	106	105	96	104	106	104	100	100	105	102	102	77	65	49	66	79	51	64	63	52	79	61	46	60	79	49	60	61	47	61	125	

Figure S2. Phylogenetic distribution of central channel components shows a marked decrease in the number of TFII members among the alveolates. Each bar represents a different component. A pool of protein sequences were selected from the 5 species then stored as a local databasein Geneious. The *Lingulodinium* transcriptome was scanned using tBLASTn at an expect E-value cutoff of e^{-25} to obtain the homologues.

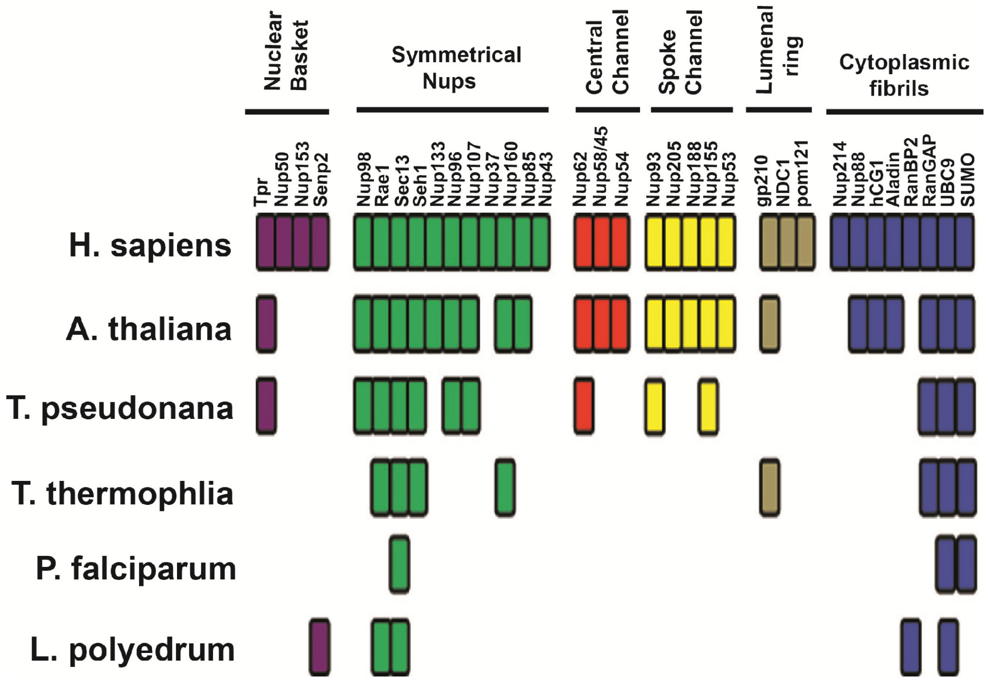

Supplement: Supplementary File 1 [file microorganisms-01-00071-s001.pdf]
